# Parental Awareness of the Preschool Orthoptics Visual Screening in Brunei-Muara District and Factors Contributing to Defaulters

**DOI:** 10.22599/bioj.349

**Published:** 2024-05-21

**Authors:** Sharimawati Sharbini, Nur Aimi Diyana Awang Damit, Ted Maddess, Siti Nurliyana Abdullah

**Affiliations:** 1 PAPRSB Institute of Health Sciences, Universiti Brunei Darussalam, Brunei Darussalam; 2 Eccles Institute of Neuroscience, John Curtin School of Medical Research (Bldg. 131), Australian National University, Canberra, ACT, 2601, Australia; 3 RIPAS Hospital, Brunei Darussalam

**Keywords:** Amblyopia, Orthoptics, Preschool, Visual Screening, Defaulters, Vision Disorders

## Abstract

**Background:**

The preschool orthoptics visual screening program began in Brunei Darussalam in 2004 to detect amblyopia, a common cause of treatable visual disorders in children. Amblyopia can be asymptomatic, easily missed, and cause permanent adverse visual consequences; hence, it is necessary to be screened. The parental role in ensuring timely visual screening is pivotal to their child’s visual well-being and educational success. This study explored parental awareness and reasons for their nonattendance.

**Methods:**

A cross-sectional study of 401 parents was conducted in the Brunei-Muara district in private kindergarten schools and maternal and child health clinics. A self-designed and self-administered questionnaire was used. Data collected was analysed using RStudio in the form of descriptive and analytic statistics.

**Results:**

The study findings showed that 52.8% defaulted their screening and there was a significant association between parental awareness and the defaulters (*p* < 0.05). Only 39.9% of parents were aware of the screening service availability, and 50.1% had not taken their children for an eye check. The most significant sociodemographic factor that influenced awareness of the importance of vision screening was parental employment status (*p* = 0.013), revealing a 4.43 times higher likelihood of default if the father was unemployed. This study found that with each additional child, parents are 1.25 times less likely to seek eye screening (*p* < 0.05).

**Conclusions:**

The main reason for nonattendance was a lack of awareness of the situation and parents believed that their children were seeing well. Mitigating child visual screening defaults requires a community-focused approach.

## Introduction

The Preschool Orthoptics Visual Screening (POVS) started in Brunei Darussalam in August 2004, aimed to detect visual disorders, particularly amblyopia, in children prior to entering primary schools. In Brunei Darussalam, this service is provided free of charge for children, regardless of nationalities and residency status of the parents and/or children. The visual system goes through a period of rapid maturation during the ‘critical period’, from birth to seven or eight years old (Hensch & Quinlan 2018), where it is extremely susceptible to visual disruption. These disorders, particularly amblyopia, can seem asymptomatic and thus are necessary to be screened for at an early age to increase the chance for better prognosis of treatment and attain a functional outcome (Atowa, Wajuihian & Hansraj 2019). The National Health Service and the United Kingdom National Screening Committee recommends vision screening in children aged between four and five years old, allowing sufficient time for intervention and treatment. The effectiveness of treatment decreases as the child ages (Hensch & Quinlan 2018).

Amblyopia refers to reduced best corrected visual acuity in one or both eyes, not attributable directly to structural abnormality of the eye or posterior visual pathways (Campos 1991), commonly causing monocular vision loss. Amblyopia can lead to permanent visual impairments and increased risk of potential blindness later in life. Early intervention is necessary to ensure better prognosis of treatment, a functional outcome and reversibility of visual impairment (Stewart et al. 2005). Globally, amblyopia affects approximately 1–4% children (Chia et al. 2010; Fu et al. 2014; Ganekal et al. 2013; Li et al. 2019; Mocanu & Horhat 2018). Amblyopia is usually caused by strabismus, astigmatism, anisometropia or bilateral high refractive errors, or physical stimulus deprivation such as by cataract or ptosis (Wright & Strube 2019).

Vision is important. Perception, cognition and learning are conveyed by vision. Any disruption can adversely affect quality of life, academic performance, physical and psychological health, and job choices. Visual impairments can decrease children’s ability to focus, read and study. Poor vision has been reported to cause children’s dropping out of school and increases the risk of being bullied (Horwood et al. 2005), negatively impacting their mental health. Visually impaired or blind adults have limited occupational choices and difficulty gaining employment (Bell & Mino 2015). This diminishes an individual’s impact on society and decreases a country’s overall productivity, thereby a decline in the economy.

Preschool visual screening by orthoptists has a higher sensitivity compared being performed by other personnel (North & Menon 2011; Wormald 1991). Orthoptists are especially trained to objectively assess all grades of vision in seemingly asymptomatic children (Barry & König 2003). There is evidence that screening delivered by appropriately trained non-orthoptic personnel can be of high quality (Garretty 2017). In Brunei, school-health nurses screen children who recognise alphabetical letters aged six and older; preschoolers are screened by orthoptists.

Parental knowledge and awareness of children’s visual health are crucial in preventing permanent vision loss. Amblyopia can present with normal vision when both eyes are open because the dominant eye compensates for the weaker amblyopic eye (Lopes-Ferreira et al. 2013). This may result in it going unnoticed and consequently late treatment. Parents of children with no family history of visual problems are less aware and less likely to seek treatment as compared to those who have positive family history (e.g., wearing spectacles) (Amiebenomo, Achugwo & Abah 2016). Lack of parental awareness and knowledge can be the reasons for defaulting from attending visual screening appointments and/or poor adherence to treatment.

The POVS program in Brunei Darussalam is offered at the Maternal and Child Health Clinics (MCHs). There are 16 MCHs distributed across the country, with eight located in Brunei-Muara, the most populated of the four districts. Children are given their POVS appointment during their routine visits (from after birth to five years old) at their allocated MCHs, in accordance with the clinics’ catchment area. Parents are reminded via text message or Short Message Service (SMS) notification a few days before the scheduled appointment. Currently, the POVS is performed exclusively by two orthoptists employed by the Ministry of Health, Brunei Darussalam. An increasing rate of defaulters would imply an increased risk for permanent adverse visual consequences. Defaulters also lead to wastage of human resources.

The prevalence of paediatric vision screening defaulters worldwide varies from as low as 13.8% to as high as 40.7% (Bottin, Waldhauser & Bertelli 2013; Hu et al. 2012; Noma, Carvalho & Kara-José 2011). Parents may default from screening for several reasons: believing young children don’t need eye checks, not prioritising children’s eye health, forgetting, work commitments, and lack of transportation (Schuster et al. 2009; Su et al. 2013; Sukati, Moodley & Mashige 2018). Parents can be in denial about the possibility of vision problems in their child, especially cases which, to them, are asymptomatic. In Brunei, 35.7% children defaulted on their POVS appointment across the Brunei-Muara district in 2017, and the rate of defaulters has been steadily increasing to 37.9% and 45.8% in 2018 and 2019, respectively (Abdullah, SN, Orthoptist, Eye Centre, RIPAS Hospital, personal communication September 6, 2020), despite attempts to remind parents a few days prior to the appointment. Defaulting appointments increases the chance of potential visual impairments and blindness later in life. This study aims to identify the factors to defaulting appointment.

Parental awareness is extremely important for children’s vision to be screened at the appropriate age by orthoptists. This study will assess the level of parental awareness of their child’s eye health, their availability, and the importance of the POVS; the study will also investigate the potential reasons for defaulters. The study’s findings aim to pinpoint opportunities for raising awareness about the POVS among parents, reducing the number of defaulters and ensuring that preventable early visual issues, particularly amblyopia, can be addressed promptly. For this study, defaulters are defined as parents or carers who have missed the screening appointment.

## Aim and Objectives

The main aim of the study is to assess parental awareness of the POVS and to identify factors contributing to defaulters. This study will (1) assess parental awareness on availability of the POVS, (2) explore parental awareness on the importance of the POVS, (3) evaluate parental awareness on self-reporting eye problems, (4) enumerate the rate of defaulters, (5) investigate potential reasons to defaulters for attending the POVS and (6) determine whether there is any significant association between defaulters and parental awareness and/or sociodemographic factors.

## Methods and Materials

### Study design

This study is a cross-sectional study that was conducted between 1^st^ December 2020 and 30^th^ April 2021. A self-administered questionnaire was constructed, validated and used to collect data (Appendix I). The target population was parents in the Brunei-Muara district at two different settings: A) private kindergarten (KG) schools, and B) maternal and child health clinics (MCHs).

Ethical approval was obtained from the joint committees of Institute of Health Sciences Research Ethics Committee (IHSREC) and Medical and Health Research Ethics Committee (MHREC) (Reference: UBD/PAPRSBIHSREC/2020/129). Prior to conducting the study, permissions were also obtained from the Private Education Section, Ministry of Education (MoE) and the Department of Health Services, Ministry of Health (MoH), Brunei Darussalam, to conduct the study at the mentioned settings. Participation was voluntary and participants could withdraw prior to submitting the questionnaires or leave the forms empty with no consequences. No identifying data was collected.

#### Inclusion criteria

- Parents of one or more children aged ≤ 5 years-old,
- Parents who can understand, read and write either English or Malay language,
- Parents of children attending private kindergarten schools,
- Parents of children attending maternal and child health clinics on child health clinic day.

#### Exclusion criteria

- Parents who already took part in pretesting and piloting phases,
- Parents who have participated in the same study in one of the targeted settings,
- Temporary caregivers (siblings, maids).
- Parents of children attending government schools. These were excluded as most children who attended government schools would be ≥5 years old (‘Brunei Darussalam Education Statistics 2013’ n.d.).

The study consisted of three phases: (1) pre-test of questionnaire, (2) pilot study and (3) main study. The questionnaire was adapted from other published similar studies (Baashar et al. 2020; Donaldson, Subramanian & Conway 2018; Raffa & Algethami, 2020) as there is no validated questionnaire available that is suitable for the objectives of this study. The items included in the questionnaire were confirmed by an expert panel to ensure content validity. The questionnaire has a total of 16 questions consisting of 3 open and 13 close ended questions, and divided into three sections:

- **Section A:** 7 questions on sociodemographic background
- **Section B:** 3 questions on parental awareness of children’s eyes health
- **Section C:** 6 questions on parental awareness of the POVS and factors that may contribute to defaulting appointments at the POVS

The questionnaire was available in both English and Malay languages and translated using the World Health Organizations guidelines (WHO n.d.).

### Phase 1: pre-test of questionnaire

The self-designed questionnaire was pretested on 10 parents, 5 from private kindergarten schools and 5 from the MCHs, to ensure clarity of the terms used. All questions were understood clearly, and no changes were made to the questionnaire.

### Phase 2: pilot study

A pilot study was conducted to test the reliability and validity of 30 parents, 15 from private kindergarten (KG) school and 15 from the MCHs. Cronbach’s Alpha was calculated to measure internal consistency reliability for the questionnaire item. The Cronbach’s Alpha coefficient was calculated to 1.03. It was suggested that in healthcare research, Cronbach’s alpha values of 0.80 or higher are generally desirable (Cortina 1993).

### Phase 3: main study

Main study was conducted after the completion of phase two.

#### Study settings

There were two settings: (A) Private kindergarten schools in Brunei-Muara district, based on the highest number of children and (B) the MCHs in the Brunei-Muara district, selected based on the most coverage. These were selected because these settings cater to children aged five years old and below. Defaulters may not show up at their allocated MCHs, thus private kindergarten schools were also selected. Government preschools were excluded as they start at five years old and older.

#### Participants

Participants were parents of (i) children attending KG1, KG2 and KG3 of private kindergarten schools, aged three, four and five years, respective, on 1^st^ January, and of (ii) children attending the MCHs on child health clinic day in the Brunei-Muara district. A parent, in this study, is defined as a primary caregiver (mother or father) of a biological or adopted child.

#### Sample size

Sample size was calculated, using Slovin’s formula (Ellen 2018), to be 400 participants, based on the total population of children aged between 0–4 years-old, N = 11,964.92 (Ministry of Health, Brunei 2017). A total of 401 completed questionnaires (Appendix I) were included; 173 from schools and 228 from the MCHs.

#### Recruitment process & data collection

Nurses in charge and principals of private kindergarten schools were the gatekeepers of MCHs and kindergarten schools, respectively. The gatekeepers liaise with the researchers to inform and invite eligible parents to the study and distribute the documents/links to questionnaire or QR code.

Parents first receive a Participant Information Sheet (PIS) describing the study and a consent form. Only those who voluntarily consent to participate go on to answer the questionnaire. Hardcopy questionnaires were distributed at the MCHs in the Brunei-Muara district and a link to the questionnaire online was distributed at the request of the schools. Only one parent per child is required to answer the questionnaire. Parents of multiple children were only required to answer once. Completed questionnaires were returned anonymously online from schools and to a designated drop box in the MCHs.

The recruitment steps and processing of both settings, (A) private kindergarten schools and (B) maternal and child health clinics, are as outlined:

##### (A) Private kindergarten schools

- Invitation/permission letter to all 48 private kindergarten schools, under the Ministry of Education, Brunei Darussalam, in Brunei-Muara districts as listed in Appendix II, was sent to the Department of Schools, Ministry of Education, through the Assistant Registrar of the Institute of Health Science (IHS), Universiti Brunei Darussalam (UBD), after obtaining ethics approval.
- Among those families who have responded and agreed to take part, students in KG1, KG2 and KG3 in participating schools were enrolled for possible study. Schools with the highest number of children were first sampled until the target sample size was reached.
- Among the selected candidates the parents of those KG1, KG2 and KG3 students were invited to participate in the study.

##### (B) Maternal and child health clinics

- Invitation/permission letters to all MCHs as listed in Appendix II were sent to the Department of Health Services, Ministry of Health, through the Assistant Registrar of the IHS, UBD, after obtaining ethics approval.
- MCHs with the most coverage will first be sampled in ascending order as listed in Appendix II until target sample size is reached.
- The MCHs agreeing to participate will invite parents attending the MCHs for appointments for their children’s care to participate in the study.

### Statistical analysis

Data was analysed using RStudio (version 4.3.1). Data was reported in the form of descriptive statistics and analytic tests, which include a t-test and a chi-square test. A *p*-value ≤0.05 is considered significant. Logistic regression analysis was used to assess for any significant association between defaulters and parental awareness on availability and importance of the POVS and/or sociodemographic background. Estimated model coefficient, standard error, t-value, *p*-value, odds ratio and 95% confidence interval are reported.

## Results

A total of 401 parents took part in this study. Table 1 shows the sociodemographic factors of the participants. Participants were predominantly females (83.0%). Most of the parents were aged 30 to 39 (62.1%), 35.9% had university education, and most (69.1%) had a range of 1–3 children. Most of the fathers (87.3%) and mothers (70.6%) were employed.

**Table 1:** Parental sociodemographic background.


	n	(%)

**Age (years)**		

≤29	57	(14.2)

30–39	249	(62.1)

40–49	92	(22.9)

≥50	3	(0.8)

**Gender**		

Male	68	(17.0)

Female	333	(83.0)

**Relationship**		

Father	68	(17.0)

Mother	333	(83.0)

**Education level**		

Primary school	26	(6.5)

Secondary school	115	(28.7)

Pre-university*	116	(28.9)

University	144	(35.9)

**Employment status (father)**		

Employed	350	(87.3)

Self-employed	31	(7.7)

Unemployed	20	(5.0)

**Employment status (mother)**		

Employed	283	(70.6)

Self-employed	15	(3.7)

Unemployed	100	(24.9)

**No. of children**		

1–3	277	(69.1)

4–6	111	(27.7)

7–9	11	(2.7)

≥10	2	(0.5)


*Pre-university refers to senior high school or an equivalent 2-year diploma.

Table 2 shows the parental awareness of (i) eye problems among their family members, (ii) awareness of the availability of the POVS, (iii) how did parents know about the POVS, (iv) awareness of the importance of the POVS, (v) if parents have taken children for eye tests and (vi) reasons for not taking children for eye tests.

**Table 2:** Parental awareness of eye problems, the POVS program and factors contributing to defaulters.


	n	(%)

**Parent had vision or eye problems**		

Yes	208	(51.9)

No	169	(42.1)

Not sure	24	(6.0)

**Immediate family members had vision or eye problems**		

Yes	276	(68.8)

No	101	(25.2)

Not sure	24	(6.0)

**Children had vision or eye problems**		

Yes	72	(18.0)

No	291	(72.6)

Not sure	38	(9.5)

**Had at least one not sure**	69	(17.2)

**No reported vision or eye problems**	4	(1.0%)

**Aware of the POVS services?**		

Yes	160	(39.9)

No	241	(60.1)

**How did you know about the POVS services?**		

Health professionals	120	(49.8)

TV or radio	26	(10.8)

Internet	22	(9.1)

Text message	7	(2.9)

Social media	23	(9.5)

Print media	11	(4.6)

Word of mouth	32	(13.3)

**Aware that POVS is offered at maternal and child health clinics or eye clinics?**		

Yes	206	(51.4)

No	195	(48.6)

**Aware of the importance of the POVS?**		

Yes	295	(73.6)

No	106	(26.4)

**Have taken child/children for eye check?**		

Yes	189	(47.1)

No	201	(50.1)

Not sure	11	(2.7)

**Reasons for not taking child/children for eye check?**		

Did not know	96	(36.8)

Did not see the need	7	(2.7)

The child was seeing well	85	(32.6)

Busy or have work commitments	15	(5.7)

No transport	2	(0.8)

Did not have someone else to look after the other children	3	(1.1)

Eye check appointment will be or is scheduled in the future	44	(16.9)

Forgot or missed the appointment	6	(2.3)

Had other appointment	3	(1.1)


*n* = number of responses.

Parental awareness refers to parents reporting known eye problems occurring within their household, including themselves, their spouse or their children. Problems may include astigmatism, hypermetropia, myopia and strabismus, cataract, and ptosis. Parental lack of awareness is categorised as ‘unaware’ or ‘not aware’ if parents reported that they, their family or their child have no eye problems. Interestingly, a higher percentage of parents (72.6%, n = 291) did not know whether their child had vision or eye problems. This was followed by almost half (42.1%, n = 169) of them not knowing if they themselves had vision or eye problems, and (25.2%, n = 101) not knowing if their spouses had eye or vision problems.

Parental awareness of the importance of the POVS and its availability at the MCHs and eyes clinics was also assessed. More than half (60.1%, n = 241) of parents did not know that the POVS existed. Parents were made aware of the POVS mainly through health professionals (49.8%, n = 120), and although SMS were sent to remind parents of their screening appointment, only 2.9% (*n* = 70) of parents were aware of the POVS through SMS. Although most parents (73.6%, n = 295) were aware of the importance of vision screening, more than half (50.1%, n = 201) have not taken their children for an eye test and a further 2.7% (n = 11) were not sure whether or not their children have had their eyes tested. The number of defaulters in this sample is categorised as the number of parents who have not taken their children for the POVS. The 11 who were unsure were given the benefit of the doubt. Hence the portion of defaulters for this study is 52.8% (n = 212). Although there were significantly more parents that are aware that the POVS is offered at the MCH clinics (*p* < 0.001), more than half chose to default, that is, to not attend the appointment.

Parents were asked to indicate the reasons for defaulting. The main reason was ‘did not know’ that their children had to be screened (36.8%, n = 96), followed by assuming ‘the child was seeing well’ (32.6%, n = 85).

Logistic regression analysis was performed to investigate significant associations between defaulters and sociodemographic background as well as between defaulters and parental awareness of the POVS (Table 3). Parents who have not taken their children to have their eyes tested are categorised as defaulters, and are included in the next analyses (n = 210, 50.1%). Sociodemographic factors that were significantly associated with parents defaulting from the POVS appointments included: unemployed parent (father) (*p* = 0.0097), POVS was 4.43 times more likely to non-attendance if the father is unemployed; parents who thought their children had no eye problems were associated with defaulting POVS (*p* = 0.05), being almost twice as likely to default; lastly, parents being unsure if their child had any eye problems was associated to defaulting (*p* = 0.001) being more than six times more likely to default POVS. Parents with more than one child were significantly less likely to default (*p* = 0.0097), and so having more children meant that parents were 1.25 times more likely to bring their children to be screened.

**Table 3:** Summary of logistic regression analyses between defaulters and sociodemographic factors.


	B	SE	t	p**	β (95% CI)*

**Education level (n)**					

Pre-university (116)	1.081	0.598	1.808	**0.071**	2.95 (0.89, 9.76)

**Employment status (father) (n)**					

Unemployed (20)	1.489	0.601	2.477	0.013*	4.43 (1.33, 14.75)

**Children**	–0.266	0.087	–2.588	0.0097*	0.80 (0.67, 0.95)

**Parent who has/had eye problems**					

No	–0.621	0.310	–2.003	**0.045**	0.54 (0.29, 1.00)

**Child/children (n) who have/had eye problems**					

No (291)	0.685	0.350	1.959	**0.050**	1.98 (0.99, 3.99)

Not sure (38)	1.845	0.55	3.360	0.001*	6.36 (2.11, 19.12)

**Aware that POVS is offered at maternal and child health clinics or eye clinics? (n)**					

Yes (206)	–1.509	0.314	–4.809	<0.001*	0.22 (0.12, 0.41)


Logistic regression showed that parents who had no concerns about their child’s eyes, or that were unsure if there were any problems with their child’s eyes, were more likely to default on their screening appointment than those who had concerns (OR = 0.54, *p* = 0.045 and OR = 6.36, *p* = 0.001, respectively). Parents who were aware that the POVS was offered at maternal and child health clinics or eye clinics were statistically associated with defaulters (OR = 0.22, *p* < 0.001) were 4.55 times less likely to default.

Table 4 summarises the outcomes of two further logistic regression models, each with a different bivariate decision variable. In the first case, the variable was whether or not the parents were aware of POVS. The second was whether parents thought the program was important or not. As in Table 3, all the variables from the 16 questions were included (except the one defining the decision variable) but in the interest of space we only report the significant variables.

**Table 4:** Summary of significant factors from the logistic regression analyses of association between parental awareness on the availability of the POVS.


PARENTAL AWARENESS OF THE AVAILABILITY OF THE POVS	*B*	SE	t	p**	β(95% CI)*

**Aware that POVS is offered at maternal and child health clinics or eye clinics? (n)**					

Yes (206)	2.595	0.317	8.198	**<0.001**	13.40 (7.11, 25.24)

**Aware of the importance of the POVS? (n)**					

Yes (295)	1.547	0.469	3.297	**0.001**	4.70 (1.84, 12.00)


*B* = estimated model coefficient, SE = standard error, t = t-value, p = p-value, *β* = Beta coefficient is the Odds or number of times greater or less and includes the *95% confidence interval for B, **p-value < 0.05 is significant.

Parents who were aware of the availability of the POVS were significantly aware that the service was offered at the MCH clinics (*p* < 0.001) and significantly recognised its importance (*p* = 0.001) (Table 4). When querying the parent’s self-reported understanding of their child’s eye problems (Question 10), those who said their child had no eye problems (*p* = 0.006) or were unsure if their child had any eye problem *(p* = 0.070) were significantly LESS aware of the importance of POVS (Table 5).

**Table 5:** Summary of significant factors from the logistic regression analyses of association between parental awareness on the importance of the POVS and defaulters.


PARENTAL AWARENESS OF THE IMPORTANCE OF THE POVS	*B*	SE	t	p**	β(95% CI)*

**Child/Children have/had eye problems (n)**					

No (291)	–1.310	0.475	–2.760	**0.006**	0.27 (0.10, 0.70)

Unsure (38)	–1.085	0.600	–1.807	**0.070**	0.34 (0.10, 1.12)


*B* = estimated model coefficient, SE = standard error, t = t-value, p = p-value, *β* = Beta coefficient is the Odds or number of times greater or less and includes the *95% confidence interval for B, **p-value < 0.05 is significant.

## Discussions

According to the WHO, 19 million children under 15 years of age across the globe are estimated to be visually impaired, and 63.2% of those impairments are caused by refractive errors and amblyopia. The goal of the POVS is to detect visual impairments as early as possible to prevent irreversible vision loss. The POVS has been demonstrated to be a potentially efficient and cost-effective method to identify visual disorders, but it would be even better with more work towards reducing default rates. Parents play a massive role in children’s health and development. Therefore, parental awareness of the POVS is crucial to allow early detection of any visual disorders.

The outcome of this study depicted that the prevalence of parents that were not aware of the POVS service was relatively high, at 60.1%, and this was unlike other studies, which have reported that majority of their participants were aware of the preschool vision-screening (Akuffo et al. 2020; Alsaqr & Masmali 2019). Remarkably, a majority of parents were aware that POVS were provided in MCH and eye clinics but have little or no knowledge of what the service is and its importance.

More than half of the parents had been made aware of the POVS service through health professionals in this study. Health professionals have been recognised as one of the main sources of information regarding the preschool vision screening (Akuffo et al. 2020). They play a major role not only in raising the awareness of the POVS but also in disseminating the information and knowledge of the POVS and its importance. No significant association was found between parental awareness of the POVS and sociodemographic background. Other studies have reported similar findings (Akuffo et al. 2020; Sukati, Moodley & Mashige 2018). Mostly, parents are the primary caregivers to preschool children. Parents make decisions for their children’s health based on their best interest as these children are not yet competent to make their own medical decisions (Grootens-Wiegers et al. 2017). Therefore, parental awareness of the POVS is very important so that any visual disorders can be detected, avoided, and treated as early as possible (Seymour 2018). This study shows that there is a significant association between awareness of the POVS and recognition of their importance. Parents who were aware of the POVS service were five times more likely to believe that POVS is important. People are more likely to adhere and comply to medical interventions such as screenings and treatments when they understand the underlying importance to such medical interventions or consequences if disregarded (Dutt 2017).

The prevalence of defaulters (nonattendance) worldwide for Children’s vision screening ranges from 13.8% to 40.7% (Bottin, Waldhauser & Bertelli 2013; Hu et al. 2012; Noma, Carvalho & Kara-José 2011). This study has reported that half of the parents have not taken their children for an eye check during preschool. In Brunei Darussalam, the MCH handbooks are made easily and readily available to mothers to be used during antenatal, perinatal, and postnatal periods. This includes information regarding the child’s vaccination programme up to five years old and the POVS. However, one of the major reasons for non-attendance was that parents claimed that they did not know that their children should be screened. This has also been reported in previous articles (Donaldson, Subramanian & Conway 2018; Noma, Carvalho & Kara-José 2011). Additionally, this study analysed that the second major reason was parents assumed that their children were seeing well. Due to the asymptomatic nature of most childhood visual disorders (Chadnova et al. 2017), parents opted not to take their children for an eye check because children’s vision may appear normal to parents (Baashar et al. 2020; Raffa & Algethami 2020). Family history of vision disorder was also significantly associated with knowledge of the importance of the POVS and attendance. This was supported in a previous study that reported children are very likely to be taken for an eye check in the presence of positive family history of vision problems (e.g., wearing spectacles) (Sukati, Moodley & Mashige 2018).

Sociodemographic characteristics were found to be important. There was a significant association between children not being brought to their screening appointment and being an only child, their parents being unemployed, being unsure if they had concerns about their child’s eyes and being unaware of the provision of POVS (Table 3). Children of parents who had university or pre-university education are less likely to become defaulters compared to those with primary school education (*p* = 0.07), and this was also reported in several studies (Baashar et al. 2020; Black, Boersma & Jen 2019). Despite the cost-free POVS service made available for all preschoolers in this country, this study observed that children of unemployed fathers were five times more likely to be defaulters. Similar findings from National Health Interview Survey observed that parents in the USA with lower socioeconomic status are less likely to have taken their children for vision-screening (Black, Boersma & Jen 2019). This study has shown that parents who have more than one child are less likely to miss eye screening appointments. Two studies reported that first time parents are likely to be less experienced with infant care as compared to those with multiple children (Bagheri, Tafazoli & Sohrabi 2016; Berhan & Gulema 2018). This study observed that parents who were aware that the POVS service is offered at MCHs or eye clinics were less likely to default (Odds ratio 0.22). In contrast, parental awareness of the POVS service itself was found to be insignificant. Although parents know of the POVS service, they may not know how, or be motivated, to access it.

The increasing rate of defaulters in Brunei Darussalam is becoming a concern to the public health. However, barriers or factors contributing to defaulters could be improved. As mentioned in this study, the majority of parents did not know or assumed that their children had good vision. One way to reduce the rate of defaulters is to improve awareness campaigns that include not just parents but the whole community by disseminating adequate information of the POVS, their importance and their existence.

## Limitations

This study only included a population from Brunei-Muara district, and so it may not be a true representation for the whole country. It was selected as it is the most populated district, and this can provide a strong preliminary indication of the population for the country. However, future studies could include populations from all four districts. For kindergarten schools, the link to the questionnaire was given through emails or QR codes and this was limited to only a few schools in view of inadequate time frame for this study. However, a different approach could be made by sharing the link through a messaging application, such as WhatsApp, that has the flexibility and ease to survey a wider target audience. Information gathered in the questionnaire may be subject to recall bias if parents have more than one child or if screening has taken place over a long period of time. In addition, the questionnaire takes an average of 15 minutes to complete and, therefore, may be long and burdensome for some parents to answer. The risk of carrying out this study also includes non-bias outcome from incomplete responses or dropouts. Incomplete responses were excluded from the study. This new questionnaire was developed and validated for the first time and was tailored to the Brunei population in both English and Malay as most of the population is bilingual. It examined parental perception and awareness of preschool vision screening, The limitations mentioned here will be evaluated in future studies.

## Conclusion

Despite reminders and rescheduling of missed appointments, the rate of defaulters is steadily increasing. Education level, employment status and number of children were associated with defaulters. Lack of parental awareness on the importance of screening at this early age group also results in an increased rate of defaulters. More efficient and effective communication efforts are required from all relevant stakeholders, including health professionals, education institutions and media to promote awareness of the POVS.

## Additional Files

The additional files for this article can be found as follows:

10.22599/bioj.349.s1 APPENDIX I. Questionnaire/Borang Kaji Selidik.

10.22599/bioj.349.s2 APPENDIX II. List of Private Kindergarten Schools (A) and Maternal and Child Health Clinics (B).
