# Supplementary material for: Parental Awareness of the Preschool Orthoptics Visual Screening in Brunei-Muara District and Factors Contributing to Defaulters
Source: Br Ir Orthopt J. 2024 May 21;20(1):154–64. doi: 10.22599/bioj.349 (PMC11122692; doi:10.22599/bioj.349)
Supplement: APPENDIX I. — Questionnaire/Borang Kaji Selidik. [file bioj-20-1-349-s1.pdf]

APPENDIX I

PARTICIPANT CODE
KOD PESERTA

QUESTIONNAIRE / BORANG KAJI SELIDIK

Parental Awareness of Preschool Orthoptics Visual Screening (POVS) in Brunei-Muara and Factors Contributing to Defaulters
Kesedaran Ibu Bapa Tentang Saringan Penglihatan Ortoptik Kanak-Kanak Prasekolah di Brunei Muara dan Faktor Penyebab Ketidakhadiran

We highly value and respect all your information provided. All the information will be confidential and anonymous / Kami sangat menghargai semua keterangan yang diberikan. Semua maklumat akan dianggap sulit dan dirahsiakan.

Have you participated in this study or answered the questionnaire? / Pernahkah awda ikut serta dalam kajian ini atau menjawab borang kaji selidik ini?

☐ **Yes** / Ya
☐ **No** / Tidak

If you choose YES, you DO NOT have to answer this questionnaire again and kindly return ALL the documents given/Jika awda pilih YA, awda TIDAK perlu menjawab borang kaji selidik ini dan sila kembalikan SEMUA dokumen yang telah diberikan.

If you choose NO, please answer ALL the questions unless where necessary and please tick one of the boxes unless stated otherwise/Jika awda pilih TIDAK, sila jawab SEMUA soalan jika perlu dan sila tandakan pada salah satu kotak melainkan dinyatakan sebaliknya.

SECTION A / BAHAGIAN A: ***Sociodemographic Background*** */ Latar Belakang Sociodemografi*

1. **Age** / Umur: __________ years old / tahun

2. **Gender** / Jantina:
   ☐ **Male** / Lelaki
   ☐ **Female** / Perempuan

3. **What is your relationship to the child?** / Apa hubungan awda dengan kanak-kanak tersebut?
   ☐ **Father** / Bapa
   ☐ **Mother** / Ibu
   ☐ **Others** / Lain-lain
   Please specify: / Sila nyatakan: ________________

4. **Education Level** / Tahap Pendidikan:
   ☐ **Primary** / Sekolah rendah
   ☐ **Secondary** / Sekolah menengah
   ☐ **Pre-University (A-Level, Technical and Vocational (Diploma, HND))/** Pra-university (A-Level, Teknikal dan Vokasional (Diploma, HND))

☐ **University (Bachelor, Master, PhD)** / Universiti (Ijazah Sarjana Muda, Ijazah Sarjana, PhD))

5. **Employment status of child's father** / Status pekerjaan bapa:
   ☐ E**mployed** / Bekerja
   ☐ **Self-employed** / Bekerja sendiri
   ☐ **Unemployed** / Tidak bekerja

6. **Employment status of child's mother** / Status pekerjaan ibu:
   ☐ E**mployed** / Bekerja
   ☐ **Self-employed** / Bekerja sendiri
   ☐ **Unemployed** / Tidak bekerja

7. **Number of children** / Jumlah bilangan anak**:** __________

SECTION B / BAHAGIAN B: ***Parental Awareness of Children's Eyes Health*** / *Kesedaran Ibu Bapa Tentang Kesihatan Mata Kanak-Kanak*

For questions 8–10/ Bagi Soalan 8-10,
Vision or eye problems refer to/ Masalah penglihatan atau masalah mata bermaksud**:**

- **Amblyopia (lazy eye) /** ambliopia (mata malas)
- **Refractive error (astigmatism, far-sightedness, short-sightedness) /** rabun penglihatan (rabun silau, rabun jauh, rabun dekat)**,**
- **Squint /** Mata Juling**,**
- **Cataract /** Utik,
- **Ptosis** / Kelopak mata kuyu.

8. **Have you ever had any vision or eye problems?** / Pernahkah awda mempunyai masalah penglihatan atau mata?
   ☐ **Yes** / Ya
   ☐ **No** / Tidak
   ☐ **Not sure** / Tidak pasti

9. **Has/Have any of your immediate family member(s) (e.g., spouse, parents, siblings) had any vision or eye problems?** / Pernahkah keluarga terdekat awda (seperti pasangan, ibubapa, adik beradik) mempunyai masalah penglihatan atau mata?
   ☐ **Yes** / Ya
   ☐ **No** / Tidak
   ☐ **Not sure** / Tidak pasti

10. **Does/Do any of your child/children have any vision or eye problems as mentioned above?** / Adakah anak-anak awda mempunyai masalah penglihatan atau mata seperti yang disebutkan di atas?
    ☐ **Yes** / Ya
    ☐ **No** / Tidak
    ☐ **Not sure** / Tidak pasti

SECTION C / BAHAGIAN C
Parental Awareness of the Preschool Orthoptics Visual Screening and Factors Contributing to Defaulters / *Kesedaran Ibu Bapa Tentang Saringan Penglihatan Ortoptik Kanak-Kanak Prasekolah dan Faktor Penyebab Ketidakhadiran*

11. **Are you aware of the Preschool Orthoptics Visual Screening services that are offered for children between the ages of 3 ½ to 5 years old?** / Adakah awda menyedari mengenai perkhidmatan Saringan Penglihatan Ortoptik Kanak-Kanak Prasekolah yang ditawarkan untuk kanak-kanak berumur diantara 3 ½ sehingga 5 tahun?
    - ☐ **Yes** / Ya
    - ☐ **No** / No

Answer questions 12 if you choose YES to question 11 / Jawab soalan 12 jika awda memilih YA kepada soalan 11

12. **How did you know about Preschool Orthoptics Visual Screening?** / Bagaimana awda mengetahui tentang Saringan Penglihatan Ortoptik Kanak-Kanak Prasekolah?
    - ☐ **Health professionals** / Profesional kesihatan
    - ☐ **TV or Radio** / TV atau Radio
    - ☐ **Internet** / Internet
    - ☐ **Text message** / Pesanan ringkas
    - ☐ **Social Media (e.g., Facebook, Twitter, Instagram, Tiktok** / Media sosial (seperti Facebook, Twitter, Instagram, TikTok)
    - ☐ **Print Media (e.g., Newspapers, leaflets)** / Media bercetak (seperti suratkhabar, risalah)
    - ☐ **Word of mouth (e.g., family, friends)** / Perbualan (seperti keluarga, kawan)
    - ☐ **Others** / Lain-lain
      Please specify: / Sila nyatakan: ____

13. **Are you aware of that the Preschool Orthoptics Visual Screening is offered at the Maternal and Child Health Centres or eye clinics?** / Adakah awda menyedari bahawa Saringan Penglihatan Ortoptik Kanak-Kanak Prasekolah ditawarkan di Klinik Kesihatan Ibu dan Anak atau klinik mata?
    - ☐ **Yes** / Ya
    - ☐ **No** / Tidak

14. **Are you aware of the importance of the Preschool Orthoptics Visual Screening?** / Adakah awda menyedari tentang kepentingan Saringan Penglihatan Ortoptik Kanak-Kanak Prasekolah?
    - ☐ **Yes** / Ya
    - ☐ **No** / No

15. **Have you taken your child/children for an eye check between ages of 3 ½ to 5 years old?** / Pernahkah awda membawa anak-anak awda untuk melakukan pemeriksaan mata semasa berumur diantara 3 ½ sehingga 5 tahun?
    - ☐ **Yes** / Ya
    - ☐ **No** / Tidak
    - ☐ **Not sure** / Tidak pasti

Answer questions 16 if you choose TIDAK to question 15 / Jawab soalan 16 jika awda memilih TIDAK kepada soalan 15.

16. **What were the reasons for not taking your child/children for an eye check?** (**You can tick more than one)** / Apakah sebab untuk tidak membawa anak-anak awda untuk melakukan pemeriksaan mata? (Awda boleh memilih lebih dari satu)
    - ☐ **Did not know** / Tidak tahu
    - ☐ **Did not see the need** / Tidak melihat kepentingannya
    - ☐ **The child was seeing well** / Anak boleh melihat dengan baik
    - ☐ **Busy or have work commitments** / Sibuk atau mempunyai komitmen kerja
    - ☐ **No transport** / Tidak ada pengangkutan
    - ☐ **Did not have someone else to look after the other children** / Tidak ada orang lain untuk menjaga anak-anak yang lain
    - ☐ **Forgot or missed the appointment** / Lupa atau terlepas perjanjian
    - ☐ **Had other appointment** / Mempunyai perjanjian yang lain
    - ☐ **Others** / Lain-lain

      Please specify: / Sila nyatakan: _______

____
