# Supplementary material for: Parental Awareness of the Preschool Orthoptics Visual Screening in Brunei-Muara District and Factors Contributing to Defaulters
Source: Br Ir Orthopt J. 2024 May 21;20(1):154–64. doi: 10.22599/bioj.349 (PMC11122692; doi:10.22599/bioj.349)
Supplement: APPENDIX II. — List of Private Kindergarten Schools (A) and Maternal and Child Health Clinics (B). [file bioj-20-1-349-s2.pdf]

APPENDIX II – List of Private Kindergarten Schools (A) and Maternal and Child Health Clinics (B)

(A) Kindergarten schools

1. Jigsaw Playschool
2. Joyful Kids Montessori School
3. Little Calips School
4. Nusa Laila Puteri School Cawangan Tanjung Bunut
5. Rising Star Child Development
6. Sekolah Sinaran Pelangi
7. Sekolah Taman Asuhan Pertiwi
8. Sekolah Taman Didikan Anak-Anak Polis Dirajja Brunei
9. Seri Mulia Sarjana School Cawangan Kampung Santul
10. Sekolah Luqman Darussalam
11. Sekolah Wonderkidz
12. Sekolah Syamde Bright Kids Kindergarten
13. Al-Falaah School Cawangan Ban 5
14. Al-Falaah School Cawangan Sungai Akar
15. Bakti Dewa School Cawangan Jerudong
16. Bakti Dewa School Cawangan Berakas
17. Bright Jigsaw International School
18. DES School
19. Fairview School
20. Freda Radin School
21. Sekolah Rendah ‘Iqra
22. Jigsaw Primary School
23. Learning Tree School
24. Nusa Laila Puteri School Cawangan Kiulap
25. Nusa Laila Puteri School Cawangan Sungai Buloh
26. Sekolah Alif Cawangan Kampung Tungku
27. Sekolah Cahaya Bina Insan
28. Sekolah Cemerlang Abejess
29. Sekolah Kesuma Mekar, Kg. Rimba
30. Sekolah PGGMB Sungai Akar
31. Sekolah Sinaran Mas
32. Sekolah Tadikan Jaya Datin Hajah Malai Rogayah
33. Sekolah Tunas Jaya PGGMB Cawangan Lambak
34. Sekolah Tunas Jaya PGGMB Cawangan Madang
35. Stella’s School
36. Sunshine School
37. Tangga Gemilang School
38. Tinkerbell Learning School
39. Sekolah Riverside, Kg Sg Matan
40. Sekolah Riverside, Kg Masin
41. Chung Hwa Middle School
42. Sekolah St. Andrew

43. Sekolah Yayasan Sultan Haji Hassanal Bolkiah
44. St. George;s School
45. Miftah An-Nur Islamic International School
46. Seri Mulia Sarjana International School
47. The International School Cawangan Sungai Hanching
48. Jerudong International School

(B) Maternal and Child Health Clinics

1. Berakas Health Centre
2. Pengiran Anak Puteri Hajah Muta-Wakkilah Hayatul Bolkiah (PAPHMWHB), Rimba Health Centre
3. Jubli Perak, Sengkurong Health Centre
4. Muara Health Centre
5. Pengiran Anak Puteri Hajah Rashidah Sa’adatul Bolkiah (PAPHRSB) Sungai Asam Health Centre
6. Pengakalan Batu Health Centre
7. Jubli Emas, Bunut Health Centre
8. Sungai Besar Health Clinic
